# Chicago Medical Society

**Published:** 1866-12

**Authors:** 


### PROCEEDINGS OF MEDICAL SOCIETIES.

### CHICAGO MEDICAL SOCIETY.

### SYPHILITIC CARIES OF ULNA.

Dr. Amerman exhibited the lower third of the ulna, which he had removed from a young man at the County Hospital. The patient had contracted syphilis six years ago, the symptoms of which were so far relieved by his medical attendants, that he enjoyed perfect health for a long time, with no indication of the disease, except slight ulcerations of the throat. During the war he was in active service, which much impaired his health. Some months ago, an abscess formed at the lower part of the left arm, which opened spontaneously. On entering the hospital six weeks since, there was considerable enlargement of the arm, with fistulous openings and sinuses leading to the ulna. Resection of the lower third of the ulna was performed yesterday. After removal, the bone was found necrosed through its whole thickness, presenting all the usual characteristics of that affection.

### SPONTANEOUS DISCHARGE OF A LARGE PORTION OF THE TIBIA.

Dr. Amerman also exhibited a piece of bone six inches in length, from the tibia of a young boy, who had suffered from typhoid fever. During convalescence, periostitis occurred, which caused the separation of a large piece of bone. Although the case was observed from time to time for a year and a half, no surgical operation would be permitted by the parents of the patient. At the end of this time the piece of bone, which had protruded from the limb, for some months, actually came away spontaneously.

### SYPHILITIC SYNOVITIS.

Dr. C. G. Smith described a case of synovitis of the knee joint, as the apparent symptom of syphilis, with rupia and ulceration of the throat. There was no known direct cause of the synovitis. The use of mercurials and iodide of potash apparently relieved the affection of the joint, as also the eruption and ulceration of the throat.

Dr. Seibert had observed several cases, which induced him to believe that an abuse of mercury in syphilis tended to produce destruction of the cancellated portion of bones, rather than of the shaft.

### DISEASE OF THE HEART AND KIDNEYS.

Dr. Ross exhibited the heart and kidneys removed from a patient, who had recently been at the County Hospital on three different occasions for disease of the heart.

The patient, aged 22 years, colored, had suffered two years before from acute rheumatism. After recovery, he had experienced more or less dyspnœa and palpitation.

During the first two attacks, which brought him to the hospital, he suffered from ascites and general anasarca, with labored respiration. There was a very distinct blowing sound with the systole of the heart, particularly marked at the apex. There was also a slight murmur with the second sound of the heart, heard more plainly over the base. In both of these attacks, the symptoms were relieved by the use of diuretics and an occasional hydrogogue cathartic.

Four days previous to his death, he again returned to the hospital with much aggravated symptoms. At the autopsy three gallons of serous fluid were removed from the abdomen.

The lungs were normal. The heart here presented weighed ℥xxi. There was marked thickening of the mitral valves, one of which was drawn toward the walls of the heart, by an abnormal attachment, producing an insufficiency. The semi-lunar valves of the aorta were quite normal, with the exception of a minute opening through the base of one of them.

The kidneys were enlarged and congested. On the surface of one of them, a peculiar deep, irregular cicatrix, about 1½ inches long and an inch wide.

### CIRRHOSIS WITH TUBERCULOUS PERITONITIS.

Dr. Ross also exhibited the liver and a part of the mesentery of a patient who had been under his care at the hospital. The symptoms during the illness were tenderness over the region of the liver, ascites, and for a time anasarca of the lower limbs. During the close of the illness there was paralysis of the legs.

At the autopsy, the liver was found in a condition of cirrhosis, small, and weighing only $2\frac{1}{2}$ lbs., it being easily broken down.

The upper portion of the lungs were studded with miliary tubercles, which had produced a small cavity at the apex of the lung. During the sickness there had been no cough or difficulty of breathing.

The whole peritoneum was found roughened by the presence of innumerable whitish elevations, containing minute deposits of tuberculous matter. The appearance, as presented by the portion of mesentery exhibited to the Society, was very remarkable.

### SINGULAR CASE OF PYELITIS, RESULTING FROM PERITONITIS.

Dr. Ross exhibited two most interesting specimens of pyelitis, the result of the products of inflammation of the peritoneum. At the autopsy, there was found within the abdomen a considerable quantity of dark fluid, containing flakes of fibrin and pus. The peritoneum of the walls of the abdomen, as well as that covering the abdominal organs, was covered with a thick mass of organized lymph, in the meshes of which were deposits of pus.

This organized deposit bound the bowels, gall bladder, liver and other organs, as it were, into one solid mass.

One of the most interesting features of the case was the peculiar condition of the kidneys. Both ureters were so compressed by bands of lymph, as almost to render them impervious. The result was a very extensive accumulation of urine in the pelvis and upper portion of the ureter of each kidney, which had so enormously dilated these portions of the organs, as to present the appearance of a very large cystic tumor. This was readily seen in one of the specimens, since a ligature had been placed around the ureter. The other kidney had been divided to show the effects of the disease upon its structure. The pelvic portions of the kidneys, presented marked evidence of inflammation; the other portions were normal.

There was extensive tuberculous deposits in the mesenteric glands.

The patient had been laboring under chronic tuberculous peritonitis for five months.

It was scarcely conceivable that the patient could have lived so long with such a serious disease.

### SINGULAR DEATH.

Dr. Bogue exhibited the heart of a young man, who had died suddenly, under peculiar circumstances. A police officer had just arrested him on a charge of theft. The young man, calling upon Almighty God to strike him dead if he was not innocent, denied his guilt, *and died upon the spot.* This strange incident was the subject of discourse in several pulpits, and was specially noticed in the public journals.

An examination revealed a hypertrophy of the left ventricle, with thickening of the aortic valves, and contraction of the space between them. The ascending aorta was considerably dilated. The other valves were in a normal condition.

### CYSTIC TUMOR OF THE OVARY.

Dr. Bogue also exhibited a uterus and appendages removed from a patient, who had died of cholera. The vagina presented traces of extensive ulceration, with dark patches, resembling gangrene. Dark fœtid fluid was found in the vagina, the cavity of the uterus, and even in the fallopian tubes.

The fallopian tubes were surrounded by thick extensive bands, evidently the products of former inflammation, which bound them to the uterus. The whole of the left, and nearly all the right ovary, were normal in size and general appearance. Attached to the right ovary and involving but a small portion of its tissue, was a multiple cystic tumor, a couple of inches in diameter. The previous history of the patient was unknown.

Dr. Bogue also exhibited a uterus with its appendages, which he removed from a woman, æt. 35, who had borne children, and who died from some obscure causes with exposure to cold.

The condition of the ovaries was of particular interest, since the surface of both of them presented a large number of small cystic tumors. Attached to the right broad ligament, was a very remarkable pedunculated tumor, spherical in form, and about a sixth of an inch in diameter. The peduncle was an inch long and not larger than a coarse horse hair.

There were also several corpora lutea, well marked, and a clot of blood in a cavity very recently occupied by an ovum.

### SANITARY REPORTS.

Dr. Davis read an interesting report on the sanitary condition of the city, giving also his personal experience in the treatment of cholera.

From the 1st of August to the 24th of October, he had been called to 205 cases, with symptoms of cholera. Of twenty-six patients, found in collapse, only two recovered.

One of these, a boy, passed through a convalescence of nearly a fortnight. During the recovery there was extensive sloughing of the lower half of both cornea, which, however, healed without visible cicatrix.

Of 77 cases seen in the stage of active vomiting and purging, 18 died and 59 recovered.

Of 102 cases with true choleraic (rice water) diarrhœa without vomiting, all recovered but one.

